# Evidence for the presence of synovial sheaths surrounding the extensor tendons at the metacarpophalangeal joints: a microscopy study

**DOI:** 10.1186/s13075-022-02841-7

**Published:** 2022-06-24

**Authors:** Yousra J. Dakkak, Bastiaan T. van Dijk, Friso P. Jansen, Lambertus J. Wisse, Monique Reijnierse, Annette H. M. van der Helm-van Mil, Marco C. DeRuiter

**Affiliations:** 1 Department of Rheumatology, Leiden University Medical Center, P.O. Box 9600, 2300 RC Leiden, The Netherlands; 2 Department of Anatomy & Embryology, Leiden University Medical Center, Leiden, The Netherlands; 3 Department of Radiology, Leiden University Medical Center, Leiden, The Netherlands; 4 Department of Rheumatology, Erasmus Medical Center, Rotterdam, The Netherlands

**Keywords:** Tendon sheath, Tenosynovium, Tenosynovitis, Peritendinitis, Metacarpophalangeal, Rheumatoid arthritis, Anatomy, Histology

## Abstract

MRI-detected inflammation around the extensor tendons of metacarpophalangeal (MCP-) joints is prevalent in RA and poses a markedly increased risk of RA development when present in arthralgia patients. Such inflammation is called ‘peritendinitis’ since anatomy literature reports no presence of a tenosynovial sheath at these tendons. However, the presence or absence of tenosynovium at these extensor tendons has never been studied. Therefore, an anatomical and histological study of extensor tendons at the MCP-joints of three embalmed human hands was performed. Immunohistochemical staining showed the presence of markers for synovial macrophages and fibroblast-like synoviocytes bordering a natural dorsal space next to the extensor tendon, suggesting the presence of a synovial lining. This implies that contrast-enhancement on MRI around extensor tendons at MCP-joints observed in early RA and pre-RA likely represents tenosynovitis and that inflammation of this synovial tissue is an early feature of RA.

**Supplementary Information:**

The online version contains supplementary material available at 10.1186/s13075-022-02841-7.

Human anatomy is one of the oldest cornerstones of medical education. It may seem surprising that advances in this field are still made. However, modern imaging techniques, such as MRI, visualize the human body in increasingly finer detail, posing new questions how to anatomically interpret what is seen [1]. For rheumatoid arthritis (RA), this applies to tenosynovitis of the hand and foot, i.e. inflammation of the synovial sheaths that surround tendons, which was recently reported to be characteristic for RA [2].

In feet of RA-patients, MRI-detected inflammation surrounding metatarsophalangeal (MTP)-tendons occurs frequently. Surprisingly, anatomical literature left it undetermined whether these tendons are surrounded by synovial sheaths. A recent anatomical study revealed that these tendons do possess synovial sheaths, both at flexor and extensor sides, implying that MRI-detected inflammation surrounding MTP-tendons in RA represents tenosynovitis [3].

Also in the hands of RA-patients, at the flexor and extensor tendons of metacarpophalangeal (MCP-) joints, MRI-detected tenosynovitis is prevalent. Moreover, in patients with arthralgia, contrast-enhancement around MCP-extensor tendons poses a high risk of RA-development [4]. According to anatomical atlases, the MCP-extensor tendons lack a synovial sheath; therefore, inflammation around extensor tendons is termed ‘peritendinitis’. The source of these anatomical images is unclear; to the best of our knowledge, the presence or absence of a synovial sheath at these extensor tendons was never studied. We hypothesized that the tissue surrounding the MCP-extensor tendons is not different from MTP-joints and thus that a tenosynovial sheath is present. Therefore, to reconsider the current anatomical information, and with the ultimate aim to improve our understanding of tissues inflamed in early RA, we performed a microscopy study of the extensor tendons at the MCP joint and performed immunohistochemical staining for markers of synovial tissue (fibroblast-like synoviocytes and macrophages).

Three embalmed human hands obtained from bodies donated for research and education were dissected. The materials studied belonged to persons (77- and 90-year-old males and a 92-year-old female) without morphological signs or known history of RA. From the first two hands, a block containing the cutis, subcutis, extensor digitorum tendon, surrounding connective tissue and underlying bone was removed at the MCP-joints, on which routine histology was performed with haematoxylin–eosin (HE) staining. From a third hand, a block of tissue was removed at the third MCP-joint without the bony element. On this specimen, next to HE, immunohistochemical staining was performed using anti-CD68 (14–0688-82, ThermoFisher, USA, 0.5 μg/ml; detection of macrophages), anti-CD55 (PA5-78,991, ThermoFisher, USA, 0.5 μg/ml; detection of fibrobrast-like synoviocytes) and anti-cadherin-11 (AF1790, R&D Systems, USA, 0.2 μg/ml; detection of fibroblast-like synoviocytes) antibodies. Methods and results are presented in more detail supplementary (S1).

Upon microscopy of the HE-stained transverse sections, we observed a thin space at the dorsal side of the extensor digitorum tendon (Fig. 1A–C). At the palmar side of the tendon, condensed connective tissue mainly consisting of collagen was present. The results of the extensor tendons at the third MCP-joint are presented, the other MCP-joints revealed a similar image. Immunohistochemical staining was performed to further characterize the connective tissue surrounding the extensor tendon (Fig. 1D). This showed expression of CD68, CD55 and cadherin-11, consistent with the presence of macrophages and fibroblast-like synoviocytes. Especially at the dorsal side of the tendon (lane 1), a thin layer positive for CD55 and cadherin-11 was detected bordering the natural space. Also CD68-positive macrophages were present in this layer. The tissue at the palmar aspect of the tendon (lane 2) has no cadherin-11 expression while CD55 was widely expressed, consistent with scattered fibroblasts. In addition, some scattered macrophages were detected.

**Fig. 1:**
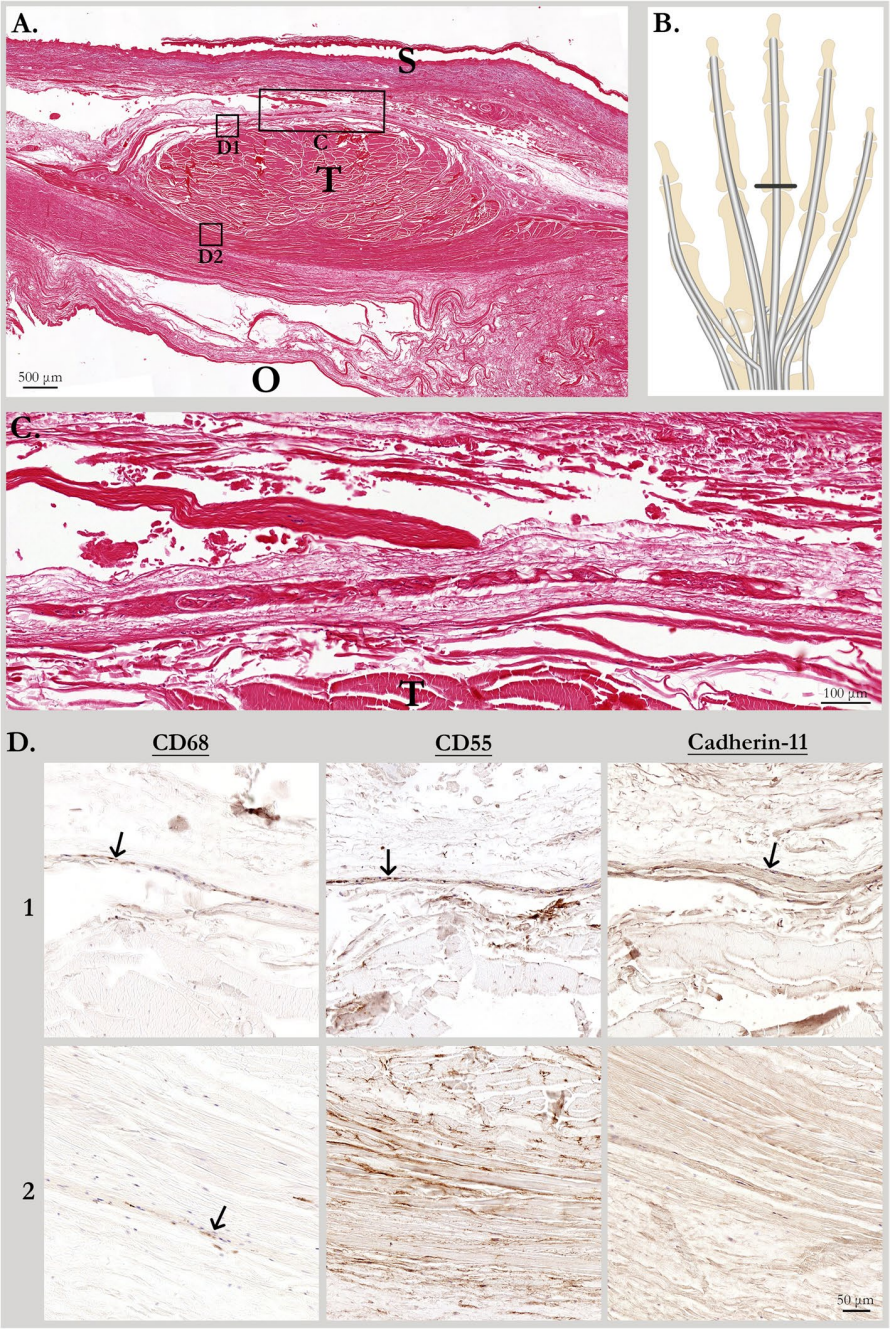
Histological and immunohistochemical evaluation of transverse sections near MCP3 through the extensor tendon. **A** HE-stained transverse section from the level indicated in **B.** T, extensor tendon; S, skin; O, location of ossa digitorum of the distal phalanx (removed from tissue block). **C** An enlargement of the HE-stained transverse section indicated by the C-box in **A**. T, extensor tendon. **D** Enlargements of adjacent transverse sections indicated by the D-boxes in A, with immunohistochemical staining for CD68, CD55 and cadherin-11. Arrows in panel D1: dorsal to the extensor tendon, a thin layer of tissue with expression of CD68, CD55 and cadherin-11 was present. Arrow in panel D2: in the tissue at the palmar aspect of the tendon some scattered macrophages were detected. There was no cadherin-11 expression, while CD55 was widely expressed, consistent with scattered fibroblasts

The results from microscopy provided evidence for presence of synovial tissue near the extensor tendons of MCP-joints, suggesting that contrast-enhancement around these tendons observed in RA represents tenosynovitis and thus inflammation of synovial tissue. An anatomical and histological study of extensor tendons from patients with active RA and controls would be the ultimate proof. Possibly, as synovial inflammation is known to involve hyperplasia and synovial thickening [5], the expression of markers used may be higher in case of active inflammation around the tendon. However, such materials are challenging to obtain. To the best of our knowledge, only a single histopathology study of tenosynovium from patients with longstanding active RA has ever been performed, which examined extensor tendons at the level of the wrist [5]. Histopathological studies on tenosynovitis, at any location, from RA or even pre-RA stages are lacking. This is a subject for future studies.

Interestingly, MRI-detected contrast-enhancement around MCP-extensor-tendons is mostly observed at the dorsal side of the tendon (Fig. 2), which is consistent with increased expression of synovial tissue markers most distinctively dorsal to the tendon. At the palmar part of the tendon the synovial lining was unclear. This might be explained by the thinness and fragility of this uninflamed tissue in healthy individuals. Additionally, the fibrous bands that interconnect the extensor-tendons to prevent subluxation whilst flexing fingers may also play a role; a dense connection may prevent the presence of an epithelium-lined space at the palmar side as observed at the flexor tendons. Remarkably, the contrast-enhancement on MRI is more flattened than around flexor tendons (Fig. 2). Presumably, this may be partly explained by the aforementioned fibrous bands causing pressure on the tendon-sheaths (Fig. 3, Supplementary Table S2 and S3). Extensor tendons are also anatomically smaller than flexor tendons and become flattened at the level of the MCP joints where they merge with the dorsal expansion of lumbrical and interosseous muscles into the extensor hood [6, 7]. Further studies with larger numbers of specimen are warranted to study anatomic variants. Nonetheless, our data suggest that contrast-enhancement around extensortendons in RA represents tenosynovitis, which may warrant modification of the classic anatomic pictures of the hand as proposed in Fig. 4. Thus, advanced imaging led to multidisciplinary research that challenged previous assumptions and revisited anatomic knowledge, resulting in a better understanding of tissues involved in RA-development. Ultimately this knowledge contributes to unravelling RA-pathophysiology.

**Fig. 2:**
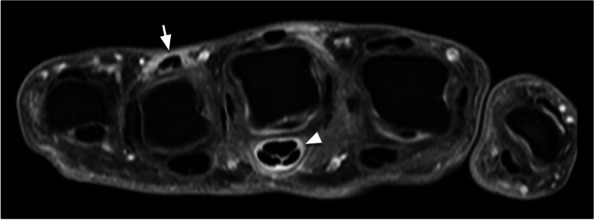
Example MR-image of extensor tenosynovitis at MCP 4 (arrow) and flexor tenosynovitis at MCP 3 (arrowhead), illustrating the oval shape of flexor tenosynovitis and the more flattened shape of extensor tenosynovitis. Axial T1-weighted fat-suppressed image after administration of gadolinium contrast (T1FS Gd) at the level of the MCPs of the right hand of a 40-year old female patient

**Fig. 3:**
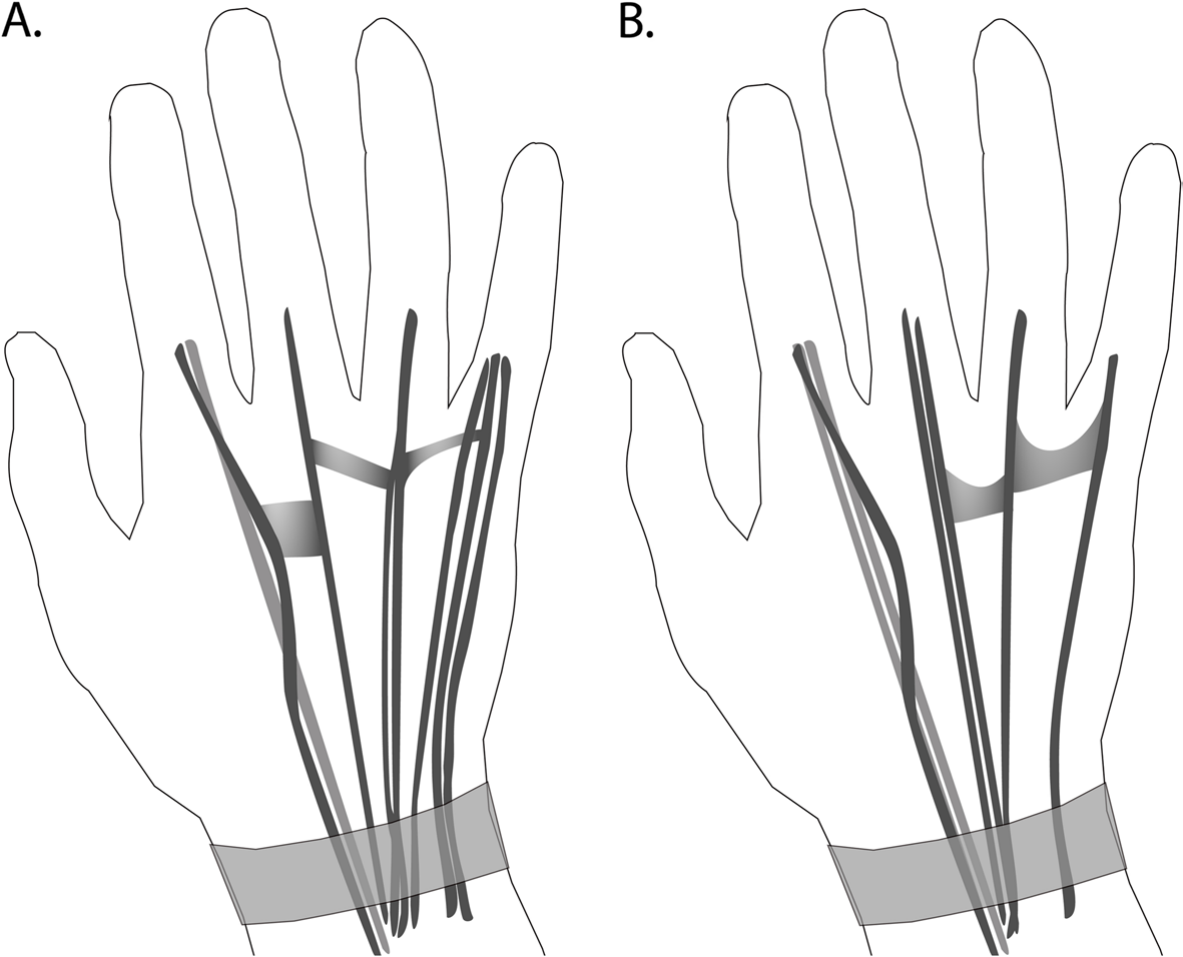
Patterns of extensor tendons and their connecting fibrous bands on the dorsum of the hand based on anatomical literature, showing the most common pattern (**A**) and one of the many anatomical variations (**B**). Figures are based on data presented in Supplementary Tables S1 and S2. **A** The most common pattern, where the index finger has a single extensor digitorum communis tendon (in dark grey) and a single extensor indices tendon (in lighter grey) that inserts at the MCP-joint ulnar to the extensor digitorum communis. The middle finger has a single extensor digitorum communis. The ring finger originates as a single extensor digitorum communis tendon that diverges into two tendons midsubstance that again merge into one right before insertion at the MCP-joint. The little finger has a single extensor digitorum communis and a double extensor digiti minimi with double insertion at the MCP-joint. The fibrous bands are present in the 2nd, 3rd and 4th intermetacarpal spaces. **B** In this variation, the index finger has a single extensor digitorum communis tendon and a double extensor indices tendon (in lighter grey) that inserts at the MCP-joint ulnar to the extensor digitorum communis. The middle finger has a double extensor digitorum communis and the ring finger a single extensor digitorum communis tendon. The little finger has a single extensor digiti minimi and an absent extensor digitorum communis. The fibrous band in the second intermetacarpal space is absent and has a Y-shape in the third and fourth intermetacarpal space (y-subtype)

**Fig. 4:**
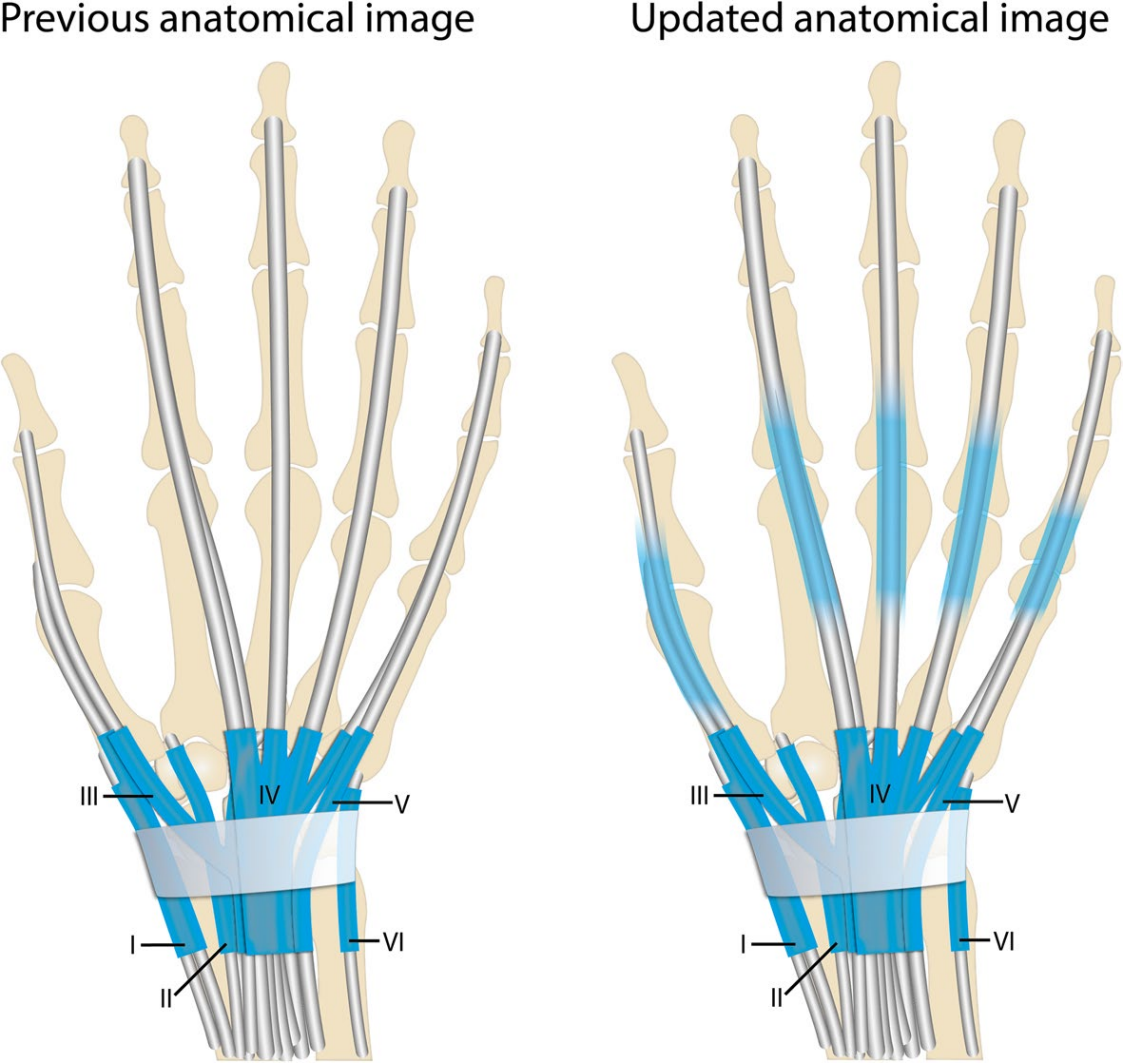
Proposal for updating anatomical textbook images of the extensor tendons of the hand based on published MRI data [8] (MCP 2–5) and on current histological findings (MCP 3). Blue represents tendon sheaths at the level of the wrist. Transparent blue represents presence of tenosynovial tissue at the MCP extensor tendons. The proximal and distal borders fade in and out on purpose, to illustrate that the area where tenosynovial tissue originates and ‘ends’ could not be deduced from this study

## Supplementary Information

**Additional file 1:** **Supplementary methods (S1). Table S2. **Patterns of extensor tendons on the dorsum of the hand according to the literature. **Table S3.** Frequency of intermetacarpal fibrous bands according to the literature.

## Data Availability

Data can be requested from the corresponding author.
