# Supplementary material for: Evidence for the presence of synovial sheaths surrounding the extensor tendons at the metacarpophalangeal joints: a microscopy study
Source: Arthritis Res Ther. 2022 Jun 24;24:154. doi: 10.1186/s13075-022-02841-7 (PMC9229148; doi:10.1186/s13075-022-02841-7)
Supplement: Supplementary file 1 — Additional file 1: Supplementary methods (S1). Table S2. Patterns of extensor tendons on the dorsum of the hand according to the literature. Table S3. Frequency of intermetacarpal fibrous bands according to the literature. [file 13075_2022_2841_MOESM1_ESM.docx]

# SUPPLEMENTARY FILE

**Supplementary methods (S1)**

The study was conducted on three F4L embalmed hands (Fix for Life BV, Leiden, The Netherlands). The specimens were obtained from bodies that were donated according to the Dutch Burial and Cremation Act to the department of Anatomy and Embryology at the Leiden University Medical Center for use in scientific research and medical education. The studies were performed at the department of Anatomy & Embryology lab of the Leiden University Medical Center in Leiden, The Netherlands.

Blocks containing the cutis, subcutis, extensor tendons located at the MCP-joint including surrounding connective tissue and part of the underlying proximal phalanx were removed from the following locations: MCP 1, 2 and 4 from donor 1, and MCP 3 from donor 2. Harvested tissues were embedded in paraffin. The paraffin blocks were transversely sliced in sections of 10 µm. The morphology of all tissues was examined by a haematoxylin and eosin (HE) staining and interpreted by two anatomists. Additionally, a block containing the extensor tendon of MCP 3 including the surrounding connective tissue but without the underlying bone was removed from donor 3 and transversely slices in sections of 10 µm. On this specimen, besides HE staining, immunohistochemical staining was performed.

After deparaffinization in xylene and rehydration, the 8 μm thick slides were incubated with 0,3%(v/v) H_2_O_2_. Then the slides were washed in phosphate-buffered saline (PBS); PBS/0,5% Tween20 (PBS-T). The primary antibodies (anti-CD55, anti-CD68 and anti-cadherin-11) were diluted in PBS-T containing 1%(m/v) BSA. The slides were incubated overnight in a humified chamber. On the next day, the slides were first incubated for 1 hour with the suitable biotinylated secondary antibody. After rinsing in PBS, PBS and PBS-T the last incubation with ABC-reagent (PK 6100, Vector Laboratories, USA) was performed. The visualisation took place with 400 μg/ml 3-3′di-aminobenzidin tetrahydrochloride (DAB; D5637, Sigma-Aldrich, USA) dissolved in tris-maleate buffer (pH 7.6) to which 20 μl H_2_O_2_ was added. Furthermore, counterstaining was done using 0.1% Mayer Haematoxylin (C0305, DiaPat, Italy) for 10 seconds, and the slides were subsequently rinsed with tap water for 10 minutes. Finally, slides were dehydrated and mounted with Entellan (1079610100, Merck, Germany).

*Primary antibodies used*

Anti CD55, PA5-78991, ThermoFisher, USA, 0.5µg/ml

Anti CD68, 14-0688-82, ThermoFisher, USA, 0.5µg/ml

Anti Cadhherin-11, AF1790, R&D Systems, USA, 0.2µg/ml

*Secondary antibodies used*

Biotin Goat anti Rabbit, BA-1000, Vector Laboratories, USA 5µg/ml

Biotin Horse anti Goat, BA-9500, Vector Laboratories, USA 5µg/ml

Biotin Horse anti mouse, BA-2000, Vector Laboratories, USA, 5µg/ml

**Supplementary results**

**Table S2.** Patterns of extensor tendons on the dorsum of the hand according to the literature.

|  | **Tendon** | **Number of tendons in dissection studies, mean % of specimen (range)*** | | | | |  |
| --- | --- | --- | --- | --- | --- | --- | --- |
|  |  | **Single tendon** | **Double tendons** | **Triple tendons** | **Quadruple tendons** | **Absent** | **Other** |
| **Index finger** | Extensor indices | 88 (77-98) | 11 (2-18) | 1 (0-7) | 0 (0) | 0.4 (0-4) |  |
|  | Extensor digitorum communis | 96 (82-100) | 4 (0-8) | 0 (0) | 0 (0) | 0 (0) |  |
| **Middle finger** | Extensor digitorum communis | 63 (51-93) | 30 (4-39) | 7 (0-19) | 0.4 (0-5) | 0 (0) |  |
| **Ring finger** | Extensor digitorum communis | 43 (12-96) | 43 (2-63) | 10 (0-22) | 3 (0-9) | 0 (0) |  |
| **Little finger** | Extensor digitorum communis | 26 (2-66) | 8 (0-25) | 1 (0-3) | 0 (0) | 20 (1-69) | Common ring- and little finger: 45 (0-93)** |
|  | Extensor digiti minimi | 15 (0-35) | 81 (63-89) | 4 (2-9) | 0.7 (0-10) | 0 (0) |  |

These numbers are based on the results and summarizing tables of Zilber et al, Dass et al and Celik et al and are from 1176 specimen in total.

*Number should be interpreted as follows: an average of 88% of specimen had a single extensor indices tendon (range 77-98%). The large variation between studies is mostly due to differences in definitions and methodology.

**Some but not all articles define a common extensor digitorum communis between the ring- and little-finger.

**Table S3.** Frequency of intermetacarpal fibrous bands according to the literature.

| **Location** | **Present, mean % of specimen (range)** |
| --- | --- |
| Second intermetacarpal space | 79 (61-87) |
| Third intermetacarpal space | 99 (96-100) |
| Fourth intermetacarpal space | 100 (100)* |

These numbers are based on the results and summarizing tables of Zilber et al, Dass et al and Celik et al and are from 204 specimen in total. Fibrous bands are also referred to as juncturae tendinae. *One study did not mention the frequency of presence of the fibrous bands at this location. It stated that analyses of the fibrous bands in the fourth intermetacarpal space is difficult because of their appearance, particularly close to the tendons, and because they often insert on the extensor apparatus of the little finger at a distant level (level of MCP-joint). As reported in literature, the fibrous bands can be classified into 3 types according to their consistency and shape (Dass et al): we have not elaborated on this.

The most common pattern of extensor tendons and fibrous bands is illustrated in supplementary Figure S1A, Supplementary Figure S1B illustrates a variation to this pattern. These figures are based on Tables S1 and S2.
